# A Study on Superior Mesoporous Activated Carbons for Ultra Power Density Supercapacitor from Biomass Precursors

**DOI:** 10.3390/ijms23158537

**Published:** 2022-08-01

**Authors:** Joon-Hyuk Bang, Byeong-Hoon Lee, Young-Chul Choi, Hye-Min Lee, Byung-Joo Kim

**Affiliations:** 1 Convergence Research Division, Korea Carbon Industry Promotion Agency (KCARBON), Jeonju 54853, Korea; lusidsoul@naver.com (J.-H.B.); bhlee@kcarbon.or.kr (B.-H.L.); youngchoi@kcarbon.or.kr (Y.-C.C.); 2 Department of Chemistry, Kunsan National University, Kunsan 54150, Korea; 3 Department of Organic Materials & Fiber Engineering, Chonbuk Natial University, Jeonju 54896, Korea; 4 Department of Carbon-Nanomaterials Engineering, Jeonju University, Jeonju 55069, Korea

**Keywords:** supercapacitor, electric double-layer capacitor (EDLC), activated carbon (AC), power density

## Abstract

A kenaf-derived activated carbon (KAC) for a high-power density supercapacitor was developed in this study through phosphoric acid activation. The N_2_/77K isothermal adsorption–desorption curve was used to estimate the textural properties of KAC based on BET and BJH and the pore size distribution based on NLDFT. The electrochemical properties of KAC were analyzed by using the coin-type cell applying 1 M SPBBF_4_/PC electrolyte, and the specific surface area and total pore volume were 1490–1942 m^2^/g and 1.18–3.18 cm^3^/g, respectively. The pore characteristics of KAC varied according to the activation temperature, and most KAC showed a mesoporous structure. As the activation temperature increased, the mesopore volume increased up to 700 °C, then decreased. The mesoporous structure of KAC resulted in a substantial decrease in the Warburg impedance as the ion diffusion resistance decreased. Hence, the specific capacitance of KAC decreased from 82.9 F/g to 59.48 F/g as the charge–discharge rate increased from 1 mA/g to 10 mA/g, with the rate of reduction at approximately 30%. The rate of reduction of KAC’s specific capacitance was 50% lower compared with commercial activated carbon; hence, KAC is a more suitable electrode-active material for high power density supercapacitors.

## 1. Introduction

With an increased global focus on addressing the challenge of environmental pollution, the interest in new energy devices that can replace petroleum has also increased [1]. Lithium ion batteries (LiB) and fuel cells serve as the main energy-storage devices based on high energy density; however, the limitation of low energy density remains a challenge [2]. Supercapacitors are characterized by semi-permanent life and high output characteristics, which makes them suitable for use as auxiliary power with LiB or fuel cells [3,4]. Supercapacitors contain a hybrid system with LiB or fuel cells to support regenerative breaking in environmentally friendly vehicles or a pitch peak and voltage dip in new renewable energy [5,6]. Numerous studies have shown that supercapacitors are beneficial as the auxiliary power in the hybrid system for generating the auxiliary output as well as securing the system’s stability and enhancing battery life [7,8,9]. In the past, supercapacitors were mainly applied in electronic devices, and the focus was on improving the energy density; however, with the recent increase in their utilization in environmentally friendly vehicles and new renewable energy, there is an urgent need to improve the power density.

In supercapacitors, the energy-storage mechanism involves the adsorption of ions to the pores of activated carbon such that the electrochemical performance relies on the characteristics of the pores of activated carbon [10]. Previous studies on supercapacitors mostly focused on improving the energy density, and as a result, the specific capacitance of supercapacitors was shown to have a strong correlation with the volume of the pore that can store the ions [11,12,13,14]. The market demand for supercapacitors with high power density has recently increased, which has prompted research that is focused on decreasing the impedance of activated carbon to increase both energy and power densities [15,16]. The electrode materials, such as the 3D building blocks, 3D activated graphene, and 3D carbon frameworks, all contain a high specific surface area that ensures outstanding energy density, whereas the high conductivity (production with nanocarbon) and low-ion diffusion resistance (high mesopore volume) were found to result in outstanding electrochemical performance (high energy density & power density) [17,18,19]. The drawbacks regarding these electrode materials, however, are the requirements for a complex synthesis, production process, and the consequential high process cost. In our previous study, a bamboo-based activated carbon for a high power density supercapacitor was produced, which had a mesoporous structure [20]. The ion diffusion resistance was found to decrease with an increase in mesopore volume. The results showed that the mesopore volume of bamboo-based activated carbon was approximately 26% higher with an approximately 11% increase in specific capacitance, compared with commercial activated carbons. To further increase the power density of supercapacitors, the mesopore volume of activated carbon should be increased further. However, if the activated carbon with a higher mesopore volume is produced by using the physical activation based on a mesopore growth mechanism via the oxidation of crystal grain boundaries, the activation yield falls below 5%, causing low economic feasibility. Thus, to develop a high-performance supercapacitor with high energy and power densities, the production of novel mesoporous activated carbons should be investigated.

Phosphoric acid activation is a manufacturing technology for producing activated carbon having a higher mesopore ratio than the physical activation method. In particular, phosphoric acid activation of biomass precursors has a pore-development mechanism in which phosphate linkages formed by crosslinking with phosphoric acid are decomposed at 723 K to form mesopores [21]. Therefore, in order to increase the mesopore ratio of the biomass-based activated carbon prepared by phosphoric acid activation, a high cellulose content required for the crosslinking reaction is required. Kenaf is a very useful biomass precursor of activated carbon with a high yield per unit area, fast growth rate, and high fixed carbon. In particular, kenaf has a high cellulose content, so it has a high utilization value as a precursor of mesoporous activated carbon by phosphoric acid activation [22].

In this study, a mesoporous kenaf-derived activated carbon (KAC) with a high specific surface area was prepared to enhance the diffusion resistance of supercapacitors. Kenaf was chosen in place of activated carbon as the precursor based on its high productivity, and phosphoric acid activation was applied to create a high specific surface area and advanced mesoporous structure. The pore-growth mechanism for KAC through phosphoric acid activation was examined by analyzing the textural properties and crystal structures. The electrochemical properties of KAC were comparatively analyzed with reference to commercial activated carbons.

## 2. Results and Discussion

**Figure 1:**
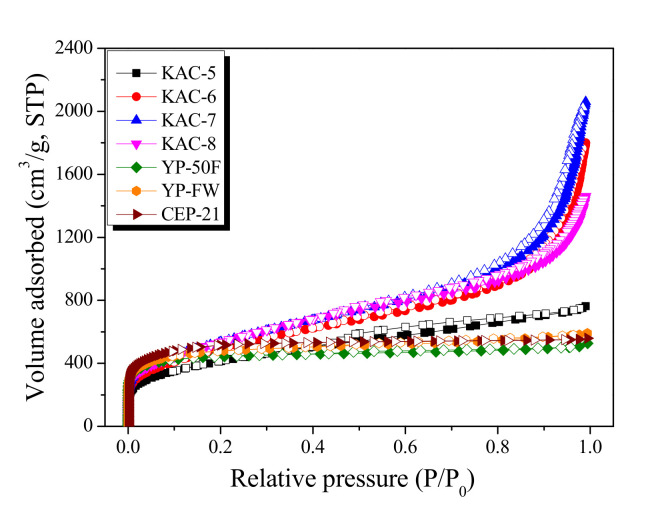
N_2_/77K isothermal adsorption-desorption curves of kenaf-derived activated carbons as a function of activation temperature.

The isothermal adsorption–desorption curve is the most effective method for analyzing the characteristics of porous carbon materials. The isothermal adsorption–desorption curves and textural properties for activated carbons (KAC and commercial activated carbons) are given in Figure 1 and Table 1, respectively.

In Figure 1, KAC and commercial activated carbons display entirely different types of isothermal adsorption–desorption curves based on the IUPAC criteria [23]. The isothermal adsorption–desorption curve for KAC showed that only KAC-5 had the type I curve, whereas all others had the type II curve. That is, the KAC activated at 500 °C mainly experiences micropore growth and the pore structure mostly comprises micropores, whereas the KAC activated at a higher temperature experiences mesopore growth and the respective pore structure comprises both micropore and mesopore. However, for commercial activated carbons (YP-50F, YP-FW, and CEP-21), the isothermal adsorption–desorption curves were all type I based on the IUPAC criteria. That is, the pore structure of commercial activated carbons mostly comprises micropores, which is presumed to be for storing a greater amount of ions to ensure a high energy density [23]. YP-FW among commercial activated carbons had the volume adsorbed fall in the range between YP-50F and CEP-21 at a relative pressure of 0.05. As the relative pressure increased, the continuous increase in the volume adsorbed led to the highest level among commercial activated carbons at relative pressure 0.99. YP-FW, therefore, showed the largest total pore volume due to high mesopore volume despite the micropore volume falling between YP-50F and CEP-21.

Table 1 shows the textural properties of activated carbon. Textural properties such as specific surface area, micropore volumes, mesopore volume, and pore size distribution of porous carbon materials are important factors to be considered when designing new materials as well as in their applications. Nitrogen adsorption has shown to be adequate to determine the textural properties of microporous and mesoporous carbon materials. However, assessing microporosity is not an easy task. As a result, several methods and models such as the Brunauer–Emmett–Teller (BET) method, the Dubinin–Radushkevich (DR) method, alpha-s plot (αs-plot), the Barrett–Joyner–Halenda (BJH) method, and the non-linear density functional theory (NLDFT) have been proposed, with each method presenting different assumptions, physical criteria, or application ranges, resulting in underestimation or overestimation of the final results in some cases. In conclusion, because KACs have a mesoporous pore structure, the two methods (DR plot and as-plot) determine the micropore volume differently depending on different assumptions, physical criteria, or application ranges in Table 1.

In Table 1, the activation yield of KAC showed a continuous fall as the activation temperature increased, which is presumed to be due to the increase in crystal oxidation with an increase in activation temperature. For KAC, the micropore volume increased until 700 °C and stabilized, but the mesopore volume increased until 700 °C and then decreased. The specific surface area and total pore volume of KAC were 1490–1940 m^2^/g and 1.18–3.18 cm^3^/g, respectively. Nonetheless, the specific surface area and total pore volume of commercial activated carbons were 1780–1950 m^2^/g and 0.83–0.91 cm^3^/g, respectively. Thus, for all except KAC-5, the specific surface area was similar to commercial activated carbons, whereas the total pore volume was two to three times higher. The results indicated that, with the exception of KAC-5, a micropore volume of KAC was similar to commercial activated carbons, whereas the mesopore volume was 5–10 times greater, so that the total pore volume became 2–3 times higher than commercial activated carbons.

The completely different pore structures between the KAC and commercial activated carbons may be attributed to the different pore growth mechanism through the activation process. The pore characteristics of activated carbons are known to be influenced by the precursor, stabilization and carbonization processes, and the activation process, the last factor having the most significant effect [24,25]. The coconut-based activated carbon (YP-50F and YP-FW) and cokes-based activated carbon (CEP-21) were each produced through the steam activation and KOH activation processes, which share the pore-growth mechanism through the crystal oxidation mediated by CO_2_ or H_2_O despite differences in the chemical activation due to intercalated metallic K. Thus, the activated carbons produced through the steam and KOH activation processes mostly display a microporous structure, whereas mesopore growth is preceded by the fall in the micropore volume, which is caused by low activation yield or pore destruction [26,27]. Therefore, it is generally very difficult to produce activated carbons with a high level of mesopores through the steam activation and KOH activation processes.

Kenaf-derived activated carbons exhibit a different mechanism of pore growth via phosphoric acid activation. The dehydration, degradation, and condensation (crosslinking) of kenaf occur due to phosphoric acid. The phosphorous compound forms an ester bond with –OH groups at 200 °C for the crosslinking in polymer chains, and thermal degradation of phosphate linkages at 450 °C or above causes the expansion of the crystal structure to increase the mesopore volume [28]. Hence, the unique pore-growth mechanism of KAC compared to commercial activated carbons is thought to underlie the high proportion of mesopores at approximately 40–60% in spite of the high activation yield.

**Figure 2:**
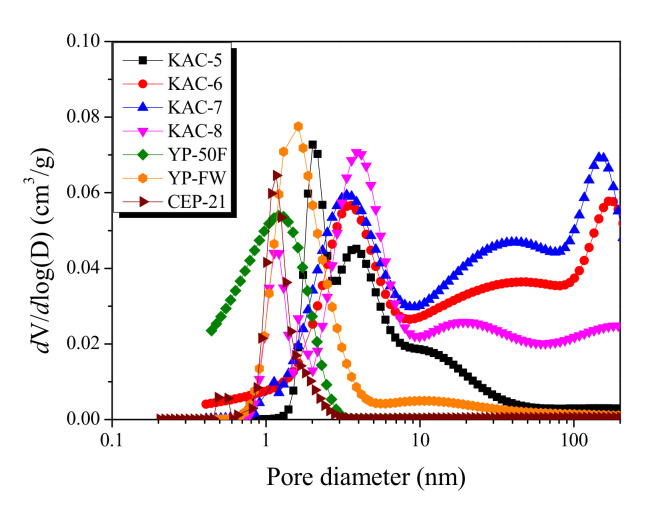
Pore size distribution curves of kenaf-derived activated carbons as a function of activation temperature.

The Non-Localized Density Functional Theory (NLDFT) is a method for monitoring the pore-size distribution in activated carbons based on the thermodynamic data. The pore-distribution curves for KAC and commercial activated carbons of EDLC are shown in Figure 2. First, compared to KAC, commercial activated carbons present narrower and smaller pore diameters on the pore-distribution curves. CEP-21, YP-50F, and YP-FW each show a curve with main pore diameters of 1.14 nm, 1.2 nm, and 1.66 nm, respectively. CEP-21, in particular, as it is formed through KOH activation, displays the narrowest pore distribution curve with the smallest pore diameter. Although YP-50F and CEP-21 have a microporous structure with pore distribution curve of <3 nm pore diameter, the mesoporous structure was most distinct for YP-FW among the commercial activated carbons with the pore growth of up to a 100-nm pore diameter.

However, a highly broad pore-distribution curve with more than two peaks was observed for KAC. The center of the peak was also observed at 3.0–4.0 nm. In line with the previously described isothermal adsorption–desorption curve of KAC, the mesopore volume with ≥10 nm pore diameter increased as the activation temperature increased up to 700 °C, then decreased. Thus, despite the similar specific surface area, KAC and commercial activated carbons showed completely different pore ranges on the micropore distribution curve, whereas KACs were found to have the pore growth with relatively larger pore diameters.

**Figure 3:**
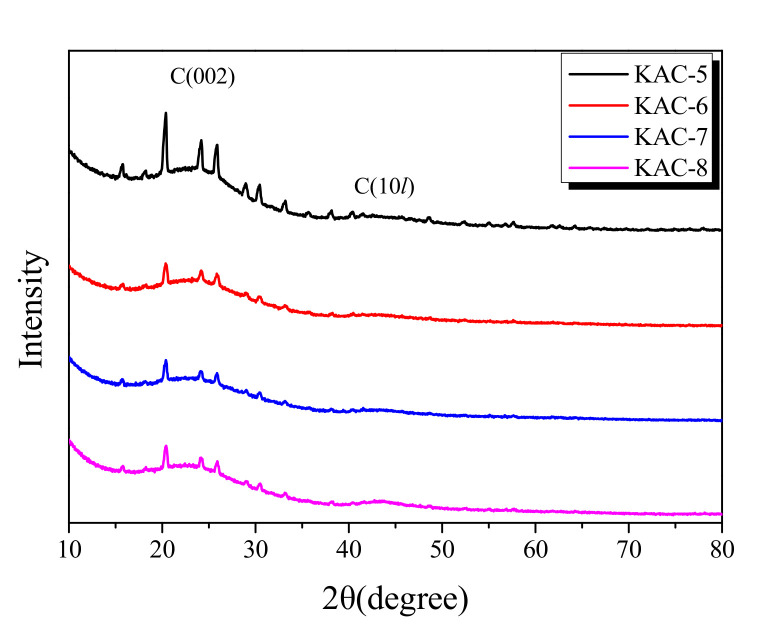
X-ray diffraction patterns of the kenaf-derived activated carbons as a function of activation conditions.

XRD is the most useful analytic method for the monitoring of the crystal structure of porous carbons. The XRD curve for KAC is presented in Figure 3. The XRD spectra agree well with the standard International Centre of Diffraction Data (ICDD) file (ICDD-PDF #411487), which confirmed the formation of crystalline structures of the graphitic carbon. The obtained peaks are indexed to the corresponding peaks for C(002), C(100), and C(101) crystal planes of graphitic carbon. However, the C(100) and C(101) crystal planes of porous carbon are marked as C(10*l*) because they are difficult to distinguish due to their low crystallinity and isotropic crystal structure. In the diffraction pattern of KAC, an Al(PO_3_)_3_ peak was observed at approximately 20° and 25°, which is considered to be formed by the reaction between phosphoric acid and an alumina tray.

**Figure 4:**
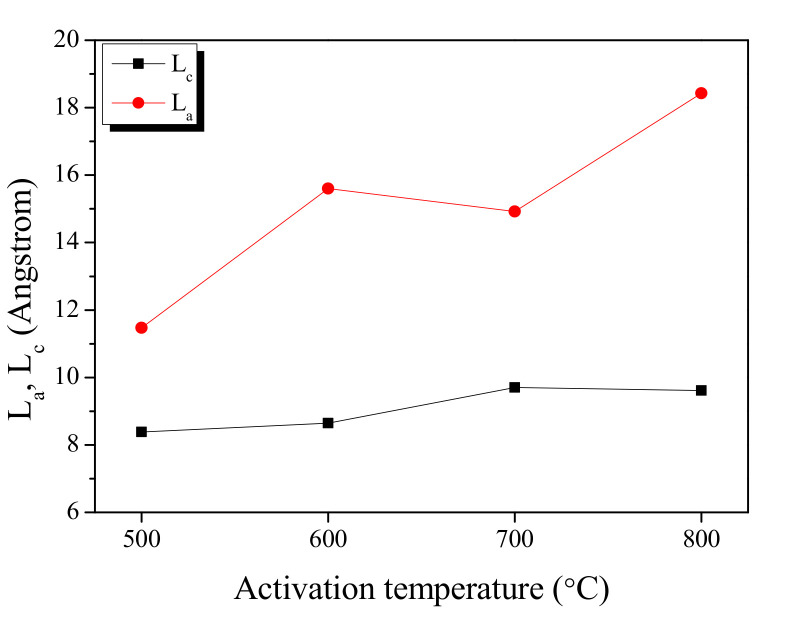
Structural characteristics of kenaf-derived activated carbons as a function of activation temperature.

**Figure 5:**
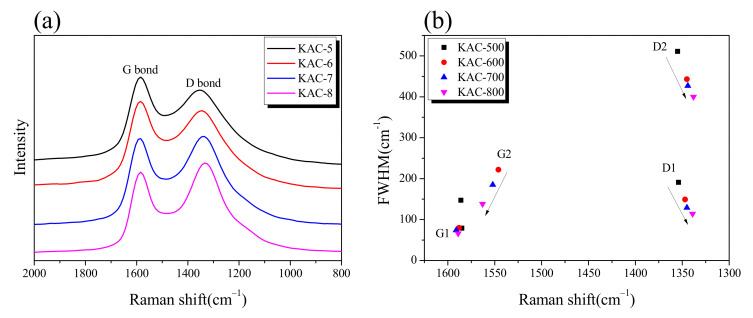
(**a**) Raman spectrum of kenaf-derived activated carbons as a function of activation temperature. (**b**) Band parameters derived from raw spectra decompositions.

The peak fitting of the XRD curve in Figure 3 is used to show the changes in the L_a_ (width) and L_c_ (height) is shown in Figure 4. The L_a_ and L_c_ increased as the activation temperature increased as shown in Figure 5. In the XRD analysis, an increase in the L_a_ of a carbon material is observed due to two reasons: (1) the crystal growth, and (2) the oxidation of a non-crystalline substance. It is a well-established fact that the crystal of carbon material grows at a high temperature [29]. The activation of KAC was mediated without carbonization after stabilization at 200 °C. Thus, the crystal growth is presumed to have occurred as the temperature increased in the activation process. As the XRD shows the mean values after statistical analyses, the oxidation of non-crystalline parts is observed with a relative increase in crystal growth [29]. As shown in Table 1, an increase in the activation temperature caused an increase in the specific surface area; this means that with the increase in activation temperature, the oxidation of non-crystalline parts increased to form micropores. As a result, the L_a_ of KAC is determined to be increased due to crystal growth, and oxidation of amorphous material at a high activation temperature.

The Raman spectrum and band parameters for KACs are shown in Figure 5. As can be seen in Figure 5a, a clear separation of G and D bands was observed with a gradual increase in ID/IG in direct proportion to the activation temperature. In our previous study, an increase in ID/IG was observed upon the oxidation of non-crystalline substances during the physical activation process [30]. Hence, the oxidation is thought to prioritize non-crystalline over crystalline substances during the chemical activation by phosphoric acid, in conformance with the previously discussed XRD result. The G1, G2, D1, and D2 calculated through peak fitting in the Raman spectrum are shown in Figure 5b. G1 is the in-plane bond stretching motion of sp2 hybridized carbon atoms pairs, D1 indicates defects/disorder, G2 indicates amorphous carbon, and D2 indicates a disordered graphitic lattice [31]. With an increase in activation temperature, a negligible change was observed in G1 across all KAC samples, whereas the FWHM decreased in G2, D2, and D1. Phosphate activation has a pore-development mechanism similar to that of physical activation in which crystal edges are oxidized by oxygen generated by the decomposition of phosphate linkages [21]. Therefore, negligible change was observed in G1, unlike G2, D1, and D2, because the crystal structure change due to the oxidation reaction of phosphoric acid activation is mainly made at the edge of the crystal. In addition, as the activation temperature increases, the oxidation due to phosphoric acid activation is likely to be more active in amorphous carbons or at the crystal grain boundaries.

**Figure 6:**
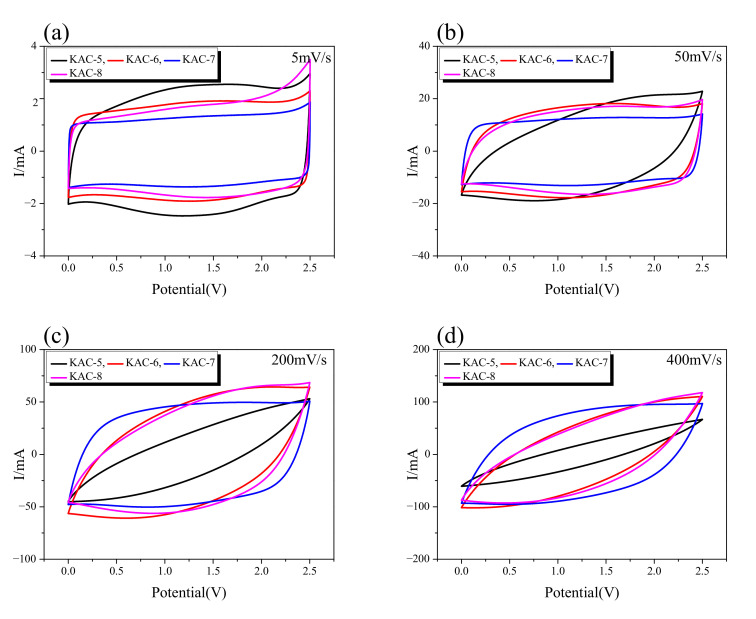
Cyclic voltammetry curves up to (**a**) scan rate 5 mV/s, (**b**) 50 mV/s, (**c**) 100 mV/s, and (**d**) 400 mV/s of AC/AC capacitors in 1 M SBPBF4 in PC electrolyte.

CV curves give a useful method for monitoring the charge–discharge behavior of EDLC. The CV curve for KAC at a 5–400 mV/s scan rate is shown in Figure 6. It is known that the CV curve for EDLC takes a rectangular shape as the most ideal form, with constant current in accordance with changes in voltage. The CV curves for all KAC samples, as shown in Figure 6, display the change to a leaf form from a rectangular form as ion-diffusion resistance increased with an increase in the charge–discharge rate. In Figure 6a, the CV curves of all KAC was observed in a rectangular form due to low ion-diffusion resistance at the slowest charge–discharge rate of 5 mV/s. The CV curve area was also highly similar across all KAC types. Thus, at slow charge–discharge rates, all KAC are presumed to have a highly similar specific capacitance.

The CV curve for KAC-5 displayed the fastest change from a rectangular to leaf form with an increase in the charge–discharge rate, whereas the leaf form was observed from 50 mV/s. Next, the CV curves for KAC-6 and KAC-8 were highly similar, whereas the leaf form was observed from 100 mV/s. The CV curve for KAC-7 stably maintained the rectangular form even at a high charge–discharge rate, whereas the leaf form was observed from 400 mV/s. It is worth noting that, at 5 mV/s, KAC-7 had the narrowest CV curve area, but at 400 mV/s, the broadest CV curve area was found; hence, despite the most outstanding levels of specific surface area and total pore volume, KAC-7 had the lowest specific capacitance at a low charge–discharge rate. At a high charge–discharge rate, however, the low ion-diffusion resistance led to the highest specific capacitance. In our previous study, the specific capacitance of EDLC was shown to be more dependent on the pore distribution required for ion storage than the specific surface area [32]. Thus, the smallest CV curve area of KAC-700 is thought to be due to low pore volume for better ion adsorption, despite the most outstanding pore characteristics. Nonetheless, the high proportion of mesopores of KAC-700 led to low ion-diffusion resistance to increase the scan rate, and to cause the highest specific capacitance.

**Figure 7:**
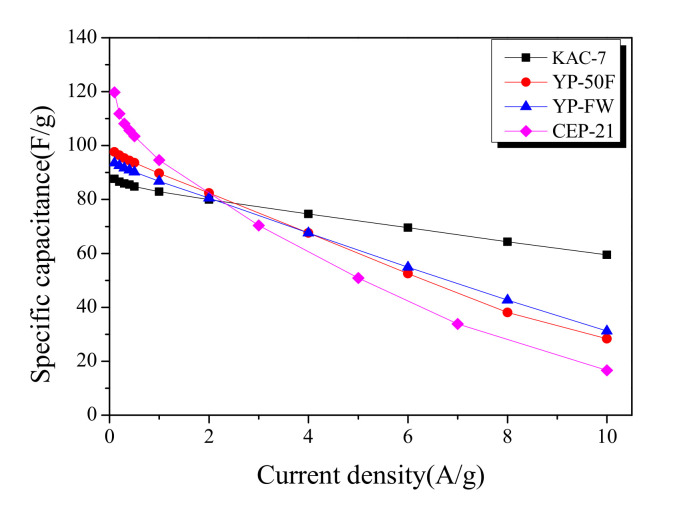
Specific capacitance curves of commercial activated carbons and kenaf-derived activated carbons with different current densities.

Galvanostatic technique allows a highly useful analytic method for monitoring the specific capacitance of activated carbons. Figure 7 shows the specific capacitance of commercial activated carbons and KAC in relation to various current densities in a 0.1–10 A/g range. At relatively low current densities of 0.1–0.2 A/g, the specific capacitance decreased from the KAC with the highest proportion of micropores, as follows: CEP-21 > YP-50F > YP-FW > KAC-700. Although the micropore volume of KAC-700 was higher than YP-50F or YP-FW, the specific capacitance was lower at 0.1–0.2 A/g current densities. In our previous study, compared to specific surface area and micropore volume, pore size distribution was shown to have greater influence on the specific capacitance [33]. As the previous pore distribution curves showed, YP-50F, YP-FW, and KAC-700 had completely different pore-distribution curves despite sharing identical specific surface area and micropore volume, and YP-50F with the pore volume indicating closer space among ions is presumed to have the highest specific capacitance.

The specific capacitance across all activated carbons showed a linear decrease with an increase in current density. The slope of decrease in specific capacitance for each activated carbon was shown to be lower for higher mesopore proportions. Thus, at ≥2.5 A/g current densities, each activated carbon acquires an altered position in the order of specific capacitance; at 10 A/g current density, the specific capacitance was in reverse order as follows: KAC-700 > YP-FW > YP-50F > CEP-21. Notably, KAC-700 was shown to maintain 67% specific capacitance as the current density increased from 0.1 A/g to 10 A/g. Thus, to increase the energy density for EDLC, a microporous structure with a pore distribution comparable to that of the ion diameter is required, and to increase the power density, a mesoporous structure is required.

**Figure 8:**
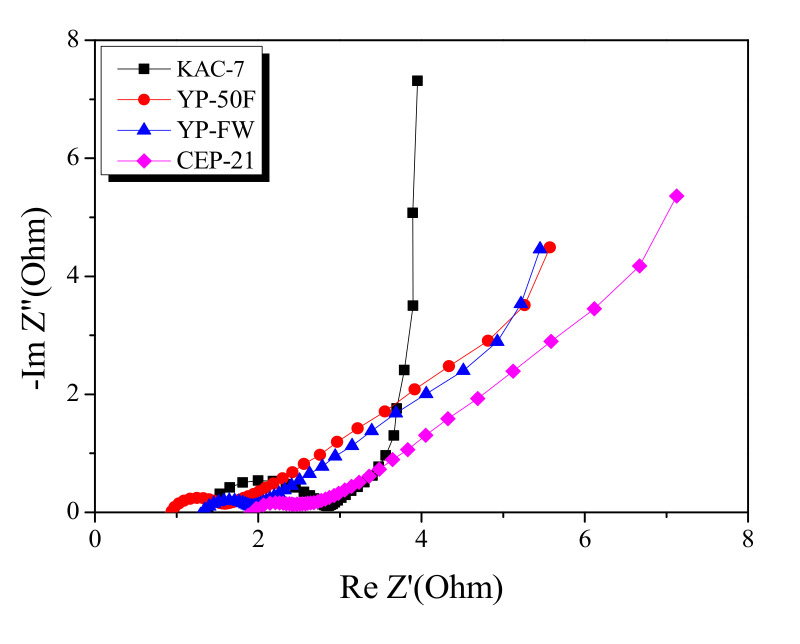
Nyquist plot of commercial activated carbons and kenaf-derived activated carbons.

Electrochemical impedance spectroscopy is the most useful analytic method for monitoring the impedance of EDLC. The Nyquist plot for KAC and commercial activated carbons are presented in Figure 8. The Nyquist plot consists of a semi-circle and a 45° slope, each indicating the charge transfer resistance and the Warburg impedance. The semi-circle diameter for KAC-7 was shown to be greater than that across commercial activated carbons. Compared to commercial activated carbons based on the precursor of high-temperature physical activation or high-level conductivity, KAC-7 is likely to show low conductivity through the activation at a relatively low temperature [33], and the resulting increase in charge transfer resistance may account for the larger semi-circle diameter.

In the Nyquist plot, the length of the 45° slope was in the following order: KAC-7 < YP-FW < YP-50F < CEP-21, with KAC-7 having the smallest slope. The result agreed with the rate of reduction of specific capacitance in the Galvanic technique result, based on the increase in current density. The Warburg impedance indicates the ion-diffusion resistance that decreases as the proportion of mesopores increases [32]. Thus, as KAC-7 has the highest mesopore volume, the reduction rate of the specific capacitance is presumed to be the lowest despite the high current density.

**Figure 9:**
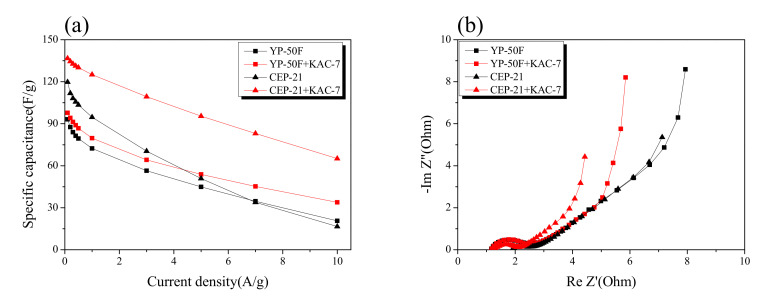
Specific capacitance and Nyquist plot of electrodes. (**a**) Specific capacitance of activated carbon and blended activated carbon electrode at different scan rates calculated from the Galvanostatic charge–discharge curve. (**b**) Nyquist plot for activated carbon and blended activated carbon electrode.

The results of the Galvanostatic technique and electrochemical impedance spectroscopy for the EDLC produced through the blending of KAC and commercial activated carbons are presented in Figure 9. After blending of KAC with CEP-21 and YP-50F in the ratio 8:2, the commercial activated carbons with a microporous structure, the consequent electrochemical behavior was monitored. In Figure 9a, the specific capacitance had a positive charge–discharge effect for commercial activated carbons through the blending with KAC. A trend of decreasing reduction rate of specific capacitance was noted after the blending of KAC with commercial activated carbons based on the high current density. At low current density, an improvement in specific capacitance was also observed.

In Figure 9b, the Nyquist plot showed an improvement in impedance after the blending of KAC with commercial activated carbons. No significant change was observed for the semi-circle of commercial activated carbons even after the blending with KAC, but the Warburg impedance was greatly improved. The KAC was thus found to have a positive effect on greatly enhancing the original EDLC performance through the blending with commercial activated carbons with a microporous structure, although it may also serve independently as an active electrode material in EDLC for high power density.

## 3. Methods and Materials

### 3.1. Materials

Kenaf and phosphoric acid were obtained from the Jeonbuk Agricultural Research & Extension Services (Iksan, Korea) and Sigma-Aldrich (St Louis, MO, USA), respectively. Kenaf was immersed in phosphoric acid for 24 h, and stabilized at 150 °C. The stabilized kenaf was placed in a self-produced alumina tubular furnace for the activation at 500–800 °C in N_2_ atmosphere for 30 min. At the end of activation, the furnace was cooled to room temperature. Subsequently, the KAC was washed with distilled water until pH7 was reached. The KAC was then pulverized to 10 μm particle size by using a ball mill (pulverisette 23, Fritsch GmbH, Idar-Oberstein, Germany) and dried in a 120 °C oven for 48 h. Subsequently, the KAC was compared with commercial activated carbons such as YP-50F, YP-FW (coconut based, steam acid activated; Kuraray, Tokyo, Japan), and CEP-21 (cokes based, KOH activated; power carbon technology, Gumi, Korea). The KAC was named KAC-activation temperature to show the experimental conditions.

### 3.2. Characteristics

The N_2_/77K isothermal adsorption-desorption curve for activated carbons was drawn by using the BELSORP-max (MicrotracBEL Corp., Osaka, Japan). For the pore characteristics, the volumes of micropore and mesopore were calculated by using the isothermal adsorption curve and Brunauer–Emmett–Teller (BET), Barrett–Joyner–Halenda (BJH), Dubinin–Radushkevich (DR), and alpha-s plot (αs-plot) equations [34,35,36,37]. For the crystal structure of activated carbons, the X-ray diffraction (RINT2000 vertical goniometer) was used in 10–90° range at 2°/min injection rate, and the structure was analyzed via Raman spectroscopy (NTEGRA; NT-MDT Instruments, Moskow, Russia) in the 500–2000 cm^−1^ range.

### 3.3. Electrochemical Test

The electrode was produced by combining KAC, carbon black (Super-P, Timcal, Bodio, Switzerland), and polytetrafluoroethylene solution (PTFE 60 wt%, Sigma-Aldrich, St Louis, MO, USA) in an 85:10:5 ratio. After mixing the electrode materials in isopropyl alcohol, a manual roller was used for the mulling, and by applying the roll press at 120 °C, a sheet electrode of 200 µm thickness was produced. The KAC electrode was attached to an etching aluminum foil of 20 µm thickness with conductive glue, then dried in a 150 °C vacuum oven for 24 h. The KAC electrode punching set to φ12 mm diameter was used to form a cell with identical cathodes and anodes. The separation membrane was composed of a φ18 mm diameter cellulose (TF4035, NKK, Hyogo, Japan); the electrolyte was 1 M spirobipyrrolidinium tetrafluoroborate (SBPBF_4_) in propylene carbonate (PC). The electrochemical properties of supercapacitors were measured at room temperature by using the Maccor 4300 battery tester (Maccor Inc., Tulsa, OK, USA) and VSP electrochemical workstation (Bio-Logic Science Instruments, Grenoble, France). For the galvanostatic charge/discharge (GCD) tests, a 0.1–10 A/g current density was applied at a voltage of 0.0–2.5 V with 20 cycles each of charge and discharge. From the 20-cycle discharge curve, the specific capacitance was calculated by using Equation (1):

(1) $$\mathrm{Cs}=\frac{\Delta T\;\times \;I}{\Delta V\;\times \;m}.$$

Here, *I* is the discharge current (A), ∆*V* is the range of constant voltage, m is the mass of the active material, and ∆*T* is the time taken for the discharge. The specific capacitance per unit mass (F/g) was calculated based solely on the mass of the active material without incorporating the binder and conductor weights. The cyclic voltammetry (CV) measurements were taken in the 0.0–2.5 V range at 5–400 mV/s scan rate. The electrochemical impedance spectroscopy (EIS) was conducted in the 300 kHz–10 MHz range at 10 mV amplitude.

## 4. Conclusions

In this study, a mesoporous kenaf-derived activated carbon was developed for the high-power density supercapacitor. Generally, KAC showed a mesoporous structure based on the kenaf decomposition, dehydration, and crosslinking via the use of phosphoric acid. The results in this study showed that kenaf is a suitable precursor in the production of activated carbon as it satisfies the ratio of the specific surface area and mesopore volume. The specific surface area and total pore volume were shown to be 1490–1942 m^2^/g and 0.83–3.18 cm^3^/g, respectively, for KAC. The specific surface area, total pore volume, and mesopore volume for KAC-7 were 1940 m^2^/g, 3.18 cm^3^/g, and 2.80 cm^3^/g, respectively. The specific surface area and micropore volume were similar between KAC and commercial activated carbons, but the mesopore volume was 2–3 times higher for KAC than for commercial activated carbons. The high mesopore volume of KAC-7 lowered the ion-diffusion resistance, exhibiting the highest output characteristics. The specific capacitance of commercial activated carbons decreased at the rate of approximately 66–68% as the current density increased from 0.1 A/g to 10 A/g, whereas the blending of KAC-7 with commercial activated carbons led to an improvement not only in the specific capacitance, but also the ion-diffusion resistance. In conclusion, the KAC produced through phosphoric acid activation exhibited more outstanding output characteristics based on the mesoporous structure, and this implied that the mesopore control on the electrode material is the key determinant in improving the supercapacitor resistance and output in the future.

## Figures and Tables

**Table 1:** Textural properties of kenaf-derived activated carbons as a function of activation temperature.

Samples	S_BET_ ^a^ (m^2^/g)	V_Total_ ^b^ (cm^3^/g)	V_Micro_ ^c^ (cm^3^/g)		V_Meso_ ^d^ (cm^3^/g)	Activation Yield ^e^(%)
			DR Method	αs Method		
KAC-5	1490	1.18	0.54	1.06	0.82	31
KAC-6	1800	2.78	0.62	1.17	2.42	30
KAC-7	1940	3.18	0.65	1.37	2.80	27
KAC-8	1940	2.25	0.64	1.36	1.86	25
YP-50F	1780	0.83	0.69	0.76	0.17	-
YP-FW	1820	0.91	0.70	0.86	0.26	-
CEP-21	2230	0.86	0.83	0.96	0.14	-

^a^ S_BET_: Specific surface area; BET method $\frac{P}{v\left(P_{0}-P\right)}=\frac{1}{v_{m}c}+\frac{c-1}{v_{m}c}\frac{p}{P_{0}}$. ^b^ V_Total_: Total pore volume; BET method. ^c^ V_Micro_: Micropore volume. ^d^ V_Meso_: Mesopore volume; BJH method r_p_ = r_k_ + t, (r_p_ = actual radius of the pore, t = thickness of the adsorbed film). ^e^ Activation yield: $\frac{\mathrm{Weight}\;\mathrm{of}\;\mathrm{activated}\;\mathrm{sample}}{\mathrm{Weight}\;\mathrm{of}\;\mathrm{carbonized}\;\mathrm{or}\;\mathrm{stabilized}\;\mathrm{sample}\;\mathrm{input}\;}\times 100$.

## Data Availability

The data presented in this study are available on request from the corresponding author.
